# Seroepidemiologic Study of Pandemic (H1N1) 2009 during Outbreak in Boarding School, England

**DOI:** 10.3201/eid1709.100761

**Published:** 2011-09

**Authors:** Sandra Johnson, Chikwe Ihekweazu, Pia Hardelid, Nika Raphaely, Katja Hoschler, Alison Bermingham, Muhammad Abid, Richard Pebody, Graham Bickler, John Watson, Éamonn O’Moore

**Affiliations:** Author affiliations: Health Protection Agency, London, UK (S. Johnson, C. Ihekweazu, P. Hardelid, K. Hoschler, A. Bermingham, R. Pebody, G. Bickler, J. Watson);; Health Protection Agency, Oxfordshire, UK (N. Raphaely, M. Abid, É. O’Moore)

## Abstract

We conducted a seroepidemiologic study during an outbreak of pandemic (H1N1) 2009 in a boarding school in England. Overall, 353 (17%) of students and staff completed a questionnaire and provided a serum sample. The attack rate was 40.5% and 34.1% for self-reported acute respiratory infection (ARI). Staff were less likely to be seropositive than students 13–15 years of age (staff 20–49 years, adjusted odds ratio [AOR] 0.30; >50 years AOR 0.20). Teachers were more likely to be seropositive than other staff (AOR 7.47, 95% confidence interval [CI] 2.31–24.2). Of seropositive persons, 44.6% (95% CI 36.2%–53.3%) did not report ARI. Conversely, of 141 with ARI and 63 with influenza-like illness, 45.8% (95% CI 37.0%–54.0%) and 30.2% (95% CI 19.2%–43.0%) had negative test results, respectively. A weak association was found between seropositivity and a prophylactic dose of antiviral agents (AOR 0.55, 95% CI 0.30–0.99); prophylactic antiviral agents lowered the odds of ARI by 50%.

## Abstract 2

TOC Summary: Prophylactic antiviral agents lower the odds of acute respiratory infection but not serologic infection.

Keywords: pandemic, influenza, A/H1N1, pandemic (H1N1) 2009, seroepidemiology, outbreak, serology, asymptomatic, prophylaxis, antiviral agents, vaccine, viruses, research

In April 2009, an influenza A subtype H1N1 virus was isolated from persons in Mexico and the United States (*1*). This virus was responsible for the first influenza pandemic of the 21st century. The first cases of pandemic influenza A (H1N1) 2009 virus infection in the United Kingdom were reported on April 27, 2009, in a married couple who returned to Scotland after visiting Mexico (*2*). Several school outbreaks were reported soon after the virus was introduced into the United Kingdom (*3*,*4*), and influenza transmission in school settings was suggested as one of the primary drivers of the spread (*5*). In the United Kingdom, boarding schools have long been recognized as a good indicator population for the onset of seasonal influenza, leading to the establishment of Medical Officers of Schools Association surveillance scheme (*6*). In many schools, a high percentage of students and staff receive a seasonal influenza vaccine each year.

On May 27, 2009, a case of pandemic (H1N1) 2009 virus infection was confirmed in a student at a large boarding school in southeastern England from a respiratory sample submitted through the Medical Officers of Schools Association scheme. Public health authorities subsequently established that an ongoing outbreak of ILI had occurred in this school in the 2 weeks before identification of the index case. Thirteen persons with onset of symptoms on or before that of the index case-patient also had positive test results, and health officials hypothesized that many unconfirmed clinical cases were also caused by infection with the emergent strain. This was the first recognized outbreak of the pandemic strain in a boarding school in the United Kingdom. In accordance with the Health Protection Agency’s (HPA) guidance at the time, postexposure antiviral prophylaxis was offered to all staff and students, and any person exhibiting symptoms of influenza-like-illness (ILI) was offered testing and prescribed a treatment dose of antiviral drugs. This outbreak and the public health control measures have been reported (*7*).

Influenza viruses are readily transmitted among residents in enclosed institutional settings (*8*). Challenge studies have suggested that one third of persons infected by influenza may be asymptomatic (*9*). In population studies, the proportion of asymptomatic influenza infections has been estimated at 50% (*10*), but whether similar proportions exist for pandemic (H1N1) 2009 is uncertain. Evidence exists regarding the effect of previous seasonal influenza vaccination on the acquisition of pandemic (H1N1) 2009 (*11*–*15*). This outbreak, with apparent transmission to many students before it was reported, provided opportunities to quantify rates of asymptomatic infection in a closed setting and study the association between exposure to the 2008–09 seasonal influenza vaccine and the use of antiviral agents with pandemic influenza (H1N1) 2009. We conducted a seroepidemiologic study in a boarding school population to describe the clinical spectrum of disease caused by the 2009 pandemic strain and to quantify the proportions of symptomatic and asymptomatic infections.

## Methods

The study population was the 1,307 students and 825 staff attending and working at the boarding school, and all were invited to participate in the study. However, because the investigation occurred during an examination period, not all students and staff were present, and the exact number staying at the school during this period is unknown. All students were boarders; some staff members lived on the school grounds, and others lived outside.

Most of the outbreak cases occurred in May 2009. Study participants were asked to complete an online questionnaire and provide a single serum sample. Samples were collected from June 11 through June 26, the last day of term. Collection of data from the online questionnaire also began on June 11 and continued until October 15.

Serologic testing by hemagglutination inhibition (HI) was carried out as previously described (*16*–*18*) at the Centre for Infections, HPA, London, using egg-grown NIBRG122 (reverse genetics derivative of A/Engl/195/2009). Serum specimens were pretreated with receptor-destroying enzyme II (Denka Seiken Co., Ltd, Tokyo, Japan), 1:4 (vol/vol), at 37°C for 19 h, followed by heat inactivation at 56°C for 1 h. The assay was performed by mixing 25 μL of virus suspension (containing 4 hemagglutinating units) with an equal volume of receptor-destroying enzyme II–treated serum, followed by 1 h incubation at room temperature, after which 25 μL of 0.5% (vol/vol) turkey erythrocytes was added to each well. Serum specimens were tested in a 2-fold serial dilution series with an initial dilution of 1:8 and ending at 1:1,024. Titers were expressed as a reciprocal of the highest serum dilution that fully prevented hemagglutination. Serum specimens with no reactivity in the first dilution (<8; considered negative) were assigned a titer of 4; serum specimens that showed titers >1,024 were assigned a numerical value of 1,024 for statistical analysis. Serologic samples were excluded from statistical analyses if a person had reported illness within 14 days of sample collection because previous data suggested that 14–21 days is required for a measurable immune response (*18*).

The online questionnaire collected data on demographic characteristics: sex; age (age groups, years: 13–15, 16–18 [students]; 20–49, >50 [staff]); symptoms; severity (self-described as mild, moderate, severe); self-reported use of antiviral drugs for treatment or prophylaxis; and self-reported 2008–09 seasonal influenza vaccination. Results from these questionnaires were subsequently linked to the serology results. Questionnaires were excluded if the person reported being away from the school during the outbreak or if symptom onset occurred after June 10, 2009.

The outcomes of interest were seropositivity and clinical cases of acute respiratory infection (ARI). Seropositivity was defined as having an HI titer >32, i.e., a titer 4× the minimum detection limit. Similar definitions have been used in population-based serosurveys in other countries (*19*,*20*) and have been shown to be specific in identifying recent infection in children (*21*). For sensitivity analysis, we refitted the final logistic regression model (below) using an alternative cutoff value of 1:8, the minimum detection limit (*22*).

A clinical case of ARI was defined as a person reporting any one of the following respiratory symptoms; runny/blocked nose, sore throat, or cough. Those reporting ARI were further subcategorized into a more specific case definition, i.e., cases of ILI, defined as a person reporting >1 of the symptoms listed above and fever. Exposures of interest were the use of antiviral drugs, prescribed for prophylaxis or treatment, and seasonal trivalent influenza vaccine in the previous year (2008–09).

We estimated the proportion of asymptomatic cases by determining the proportion of the population with positive serologic test results but no symptoms of ARI. We also estimated the attack rate for those with ARI, ILI, and positive serologic test results and their distribution according to demographic variables.

Logistic regression models were constructed to estimate the independent association of antiviral drugs and seasonal influenza vaccine and the odds of being seropositive or having an ARI. Age was included in the model as a covariate; other linear predictors were included if model fit was significantly improved (likelihood-ratio [L-R] test p<0.05). Interaction between age group and antiviral agents for prophylaxis; and seasonal influenza vaccine was tested to determine whether these associations between predictors and seropositive status and ARI differed according to age group, and therefore according to student and staff categories. If interaction was observed (i.e., the model was improved by including the interaction term), students and staff would be reported separately. A further model was also fitted for staff to investigate whether staff role and sex were associated with a seropositive status or ARI. Data analysis was carried out by using Stata version 11 (StataCorp LP, College Station, TX, USA).

Informed consent was sought from all students and their parents or guardians if students were <16 years old. Because this was a field epidemiology study conducted during an emerging pandemic and involved a novel virus with unknown clinical effects, HPA did not require formal ethical approval since any information gained was essential in illuminating the effects of the infection and indicating possible control measures.

## Results

### Sample Population

In total, 746 questionnaires were completed online, of which 695 (93.2%) met the inclusion criteria (Figure). This represented 35.9% of the 1,307 students and 27.4% of 825 staff who usually reside at the school. In total, 411 persons gave a serum sample and 353 (85.9%) were matched to a valid questionnaire (Figure). Of persons with a questionnaire and matched serologic test result, 216 were students and 137 staff, which accounts for 16.5% of the registered student population and 16.6% of the registered staff population; these 353 persons composed our final cohort (Figure).

**Figure:**
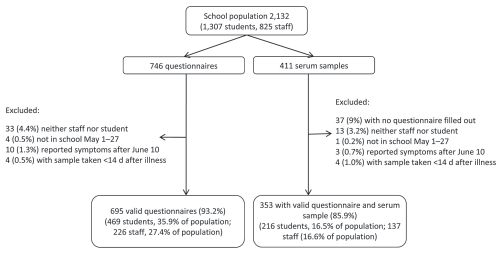
Number and proportion of boarding school staff and student populations who completed a questionnaire and had matched serologic test results, England, 2009.

### Representativeness of Study Populations

The distribution of the study population by age, sex (staff only; all students were male), occupation (staff only), self-reported illness, history of seasonal influenza vaccine in the previous year, and the use of antiviral agents is shown in Table A1. To determine whether our sample was representative of the total school population, we compared the final study population (questionnaire and matched serologic results) to those who completed only the questionnaire and to the whole school population. For staff, the proportion of men and women in the final study population was similar to the proportion that answered the questionnaire only (51.8% vs. 48.2% and 48.2% vs. 51.8%, respectively; χ^2^ p = 0.58). The proportions of students in age groups 13–15 years and 16–18 years were similar for those completing a questionnaire only (46.7% vs. 53.3%) compared to those in the matched study sample (41.7% vs. 58.3%; χ^2^ p = 0.25) (Table A1), and similar to the school’s student population. Teaching staff made up 31.6% of those of the final study population and 38.6% of the questionnaire-only population (χ^2^ p = 0.52). For persons reporting ARI, differences were significant between those who completed a questionnaire only and those who also gave a blood sample for students (37.3% vs. 48.3%; χ^2^ p *=* 0.020) and staff (10.2% vs. 30.8%; χ^2^ p*<*0.001). This selection bias could have led to an overestimation of the infection attack rate.

### Symptoms by Self-reported Illness and Serologic Test Results

For ARI, the attack rate was estimated at 35.9%, (237/661, 95% confidence interval [CI] 32.2%–39.6%) or 16.6% (110/661, 95% CI 13.9%–19.7%) by using the definition for ILI (Table A1). Of those who reported ARI and ILI, serologic test results were negative for 64/141 (45.4%, 95% CI 37.0%–54.0%) and 19/63 (30.2%, 95% CI 19.2%–43.0%) persons, respectively.

We found 143 seropositive persons, which gives an attack rate for infection of 40.5% (95% CI 35.3%–45.8%; Table 1). Of these 143 persons, 4 students did not answer the question relating to their illness status, and of the remaining 139 persons for whom illness history was available, 62 (44.6%, 95% CI 36.2%–53.3%) did not report ARI.

**Table 1:** Association of demographic characteristics, clinical illness, and interventions with study participants’ positive serologic test results during outbreak of pandemic (H1N1) 2009 at a boarding school, England*

Variable	No. participants	No. (%) with positive serologic test result	Odds ratio (95% CI)
Total	353	143 (40.5)	
Demographics			
Category			
Students	216	123 (56.9)	7.74 (4.48–13.35)
Staff	137	20 (14.6)	1
Age group, y			
13–15	90	46 (51.1)	1
16–18	126	77 (61.1)	1.50 (0.87–2.60)
20–49	71	12 (16.9)	0.19 (0.09–0.41)
>50	66	8 (12.1)	0.13 (0.06–0.31)
Sex, staff only†			
F	71	5 (7.0)	1
M	66	15 (22.7)	3.88 (1.32–11.39)
Role, staff only‡			
Nonteaching	93	6 (6.5)	1
Teaching	43	14 (32.6)	7.00 (2.46–19.90)
Clinical illness			
ARI			
No	199	62 (31.2)	1
Yes	141	77 (54.6)	2.66 (1.70–4.16)
ILI			
No	277	95 (34.3)	1
Yes	63	44 (69.8)	4.44 (2.45–8.02)
Severity, n = 153§			
Mild	81	38 (46.9)	1
Moderate and severe	59	39 (66.1)	2.21 (1.10–4.41)
Duration, d, n = 153§			
1–2	14	6 (42.9)	1
3–6	52	27 (51.9)	1.44 (0.44–4.73)
7–10	18	12 (66.7)	2.67 (0.63–11.28)
>10	23	14 (60.9)	2.07 (0.54–8.00)
Interventions			
Took antiviral drugs			
No	96	48 (50)	1
Yes	207	68 (32.9)	0.49 (0.30–0.80)
Use of antiviral drugs: PEP vs. treatment dose			
No antiviral drugs	96	48 (50.0)	
PEP dose only	187	56 (30.0)	0.43 (0.26–0.71)
Treatment dose	20	12 (60)	1.5 (0.56–4.00)
Completion of PEP course of antiviral drugs¶			
No antiviral drugs	96	48 (50.0)	1
Completed	25	9 (36.0)	0.56 (0.23–1.40)
Not completed	159	45 (28.3)	0.39 (0.23–0.67)
Seasonal influenza vaccine			
No	105	29 (27.6)	1
Yes	230	106 (46.1)	2.24 (1.36–3.69)

*Categories in which the response was missing or unknown are shown in Table A1. CI, confidence interval; ARI, acute respiratory infection; ILI, influenza-like illness; PEP, postexposure prophylaxis. †All students were male. ‡1 staff member was not included in the analysis because his occupation was unknown. §Of those that self-reported ARI. ¶Excluding those who reported taking the treatment dose of antiviral drugs.

In crude analyses, persons who reported ARI were more likely to be seropositive (62/199, 54.2%; crude odds ratio [OR] 2.66, 95% CI 1.70–4.16) than were those without illness (77/141, 29.9%). The odds of having serologic evidence of infection increased when the illness reported met the case definition for ILI (crude OR 4.44, 95% CI 2.45–8.02) (Table 1).

We also found an association between severity of reported illness and seropositivity. Overall, those reporting moderate or severe illness were more likely to be seropositive than those reporting mild illness (crude OR 2.21, 95% CI 1.10–4.41). We found no association between seropositivity and duration of illness (Table 1).

### Association between Self-reported Illness, Infection, and Interventions

Overall, fewer students reported antiviral drug use than did staff (59.4% vs. 80.0%, p<0.001). Most of those taking a treatment dose were students (16/20, 80%) (Table 2). All students completed their prophylactic course of antiviral agents vs. 91% of staff. More students reported having had the 2008–09 trivalent seasonal influenza vaccine than staff (81.1% vs. 50.0%; p<0.0001 in the matched sample).

**Table 2:** Association of demographic characteristics and interventions with study participants’ reports of ARI during outbreak of pandemic (H1N1) 2009 at a boarding school, England*

Variable	No. participants	No. (%) with ARI	Odds ratio (95% CI)
Total	695	237 (34.1)	
Demographics			
Category			
Students	469	187 (39.9)	2.53 (1.75–3.65)
Staff	226	50 (22.1)	1
Age group, y			
13–15	219	75 (34.2)	1
16–18	250	112 (44.8)	1.55 (1.06–2.28)
20–49	111	32 (28.8)	0.70 (0.42–1.15)
>50	87	18 (20.7)	0.48 (0.26–0.86)
Sex, staff only†			
F	109	24 (22.0)	1
M	117	26 (22.2)	0.98 (0.52–1.83)
Role, staff only			
Nonteaching	135	26 (19.3)	1
Teaching	85	23 (27.1)	1.48 (0.78–2.82)
Interventions			
Took antiviral drugs			
No	198	81 (40.9)	1
Yes	393	110 (28.0)	0.55 (0.38–0.79)
Use of antiviral drugs: PEP vs. treatment dose			
No antiviral drugs	198	81 (40.9)	1
Yes, PEP	352	78 (22.2)	0.40 (0.27–0.59)
Yes, treatment dose	41	32 (78.1)	4.87 (2.20–10.77)
Completion of PEP course of antiviral drugs†			
No antiviral drugs	198	81 (40.9)	1
Not completed	51	14 (27.5)	0.44 (0.17–1.15)
Completed	294	62 (21.1)	0.38 (0.22–0.65)
Seasonal Influenza vaccine			
No	216	66 (30.6)	1
Yes	425	161 (37.9)	1.35 (0.95–1.92)

*Categories in which the response was missing or unknown are shown in the Table A1. ARI, acute respiratory infection; CI, confidence interval; PEP, postexposure prophylaxis. †Excluding those who reported taking the treatment dose of antiviral drugs.

In logistic regression models for ARI and serologic status (Table 3), including age group, significantly improved the fit of the models (both L-R tests p<0.001 compared models, including only antiviral drug use and vaccination status). No evidence of effect modification was found between age group and antiviral drugs or vaccination status for either outcome (L-R test p = 0.87 and p = 0.77, respectively). Therefore, stratified models were not fitted for staff and students.

**Table 3:** Multivariable analysis of all study participants in relation to having ARI or serology-confirmed infection during outbreak of pandemic (H1N1) 2009, England*

Variable	AOR (95% CI) for ARI in questionnaire sample	AOR (95% CI) for positive test result in matched sample
Age group, y		
13–15	1	1
16–18	1.57 (0.98–2.53)	1.85 (0.95–3.60)
20–49	1.00 (0.53–1.89)	0.30 (0.12–0.73)
>50	0.66 (0.32–1.34)	0.20 (0.08–0.53)
Took antiviral drugs for PEP†		
No	1	
Yes	0.41 (0.27–0.61)	0.55 (0.30–0.99)
Seasonal Influenza vaccine		
No	1	1
Yes	1.01 (0.63–1.62)	1.81 (0.91–3.59)

*For ARI, n = 654 who completed questionnaires; for serology-confirmed infection, n = 333 who completed questionnaires and had a matched serology sample. ARI, acute respiratory infection; AOR, adjusted odds ratio; CI, confidence interval; PEP, postexposure prophylaxis. †Persons who reported taking treatment dose of antiviral agents were excluded.

Staff in the age groups 20–49 years and >50 years (adjusted ORs [AORs] 0.30 [95% CI 0.12–0.73] and 0.20 [95% CI 0.08–0.53], respectively) were less likely to have positive serologic test results than students 13–15 years of age (Table 3). This effect was not observed when ARI was used as the outcome. Weak evidence suggests that those 16–18 years were more likely to be seropositive and have ARI than those 13–15 years of age (AOR 1.85 [95% CI 0.95–3.60] and 1.57 [95% CI 0.98–2.53], respectively). Although odds of seropositivity did not increase significantly with receipt of 2008–09 seasonal influenza vaccine (p = 0.10), the point estimate was >1.

Likewise, the point estimate of the AOR for the association between taking a prophylactic dose of antiviral drugs and seropositivity was <1 (p = 0.045). In a similar model, with ARI as the outcome of interest, having received a prophylactic dose significantly reduced the odds of ARI (AOR 0.41, 95% CI 0.27–0.61).

For the staff-only models (Table 4), staff role improved the model that predicted serologic results and ARI (L-R test p <0.001 and 0.01, respectively). After staff role was taken into account, including sex as a factor did not improve the accuracy of either model and was therefore not included. For the multivariable logistic regression model, which included only staff, age groups, staff role, exposure to prophylactic dose of antiviral agents, and having received the 2008–09 seasonal influenza vaccine were considered. Teachers were more likely be seropositive than other staff members (AOR 7.47, 95% CI 2.31–24.18), and no association was found between seropositivity outcome and age, taking the prophylactic dose of antiviral drugs, or receiving the influenza vaccine (Table 4).

**Table 4:** Multivariable analysis of staff only in relation to having ARI or serology-confirmed infection during outbreak of pandemic (H1N1) 2009, England*

Variable	AOR (95% CI) for ARI in questionnaire sample	AOR (95% CI) for positive test result in matched sample
Age group, y		
20–49	1	1
>50	0.71 (0.32–1.57)	0.91 (0.26–3.14)
Role		
Nonteaching	1	1
Teaching	1.18 (0.56–2.52)	7.47 (2.31–24.18)
Took antiviral drugs for PEP†		
No	1	1
Yes	0.34 (0.15–0.77)	0.66 (0.18–2.39)
Seasonal influenza vaccine		1
No	1	0.76 (0.35–1.67)
Yes	2.36 (0.69–8.11)	2.36 (0.69–8.11)

*n = 226. ARI, acute respiratory infection; AOR, adjusted odds ratio; CI, confidence interval; PEP, postexposure prophylaxis. †Persons who reported taking the treatment dose of antiviral drugs were excluded.

When the final logistic models were refitted by using the minimum detection limit (>1:8) to define seropositive status, this change made little difference to the point estimates of the ORs for antiviral drug use, age group, or seasonal vaccine. For the staff-only model, using a cutoff value >1:8 changed the point estimates for taking antiviral agents and age groups (>50 vs. 20–49 years) to >1; however, neither linear predictor was significantly associated with the outcome with either cutoff value.

## Discussion

This study describes the seroprevalence of infection with the pandemic (H1N1) 2009 virus in an enclosed institutional environment and provides evidence of widespread infection among both students and staff before the outbreak became evident to public health authorities. Attack rates for infection were estimated as 40.5% by serologic testing and as 34.1% by clinical illness (ARI). An estimated 44.7% serology-positive persons did not report symptoms of ARI, which agreed with previous findings (*10*,*23*). No significant association was found between seropositivity and prophylaxis with antiviral drugs, although some evidence showed that it reduced the odds of ARI. The point estimate of the AOR indicated nonsignificant increased odds of infection (indicated by serologic results) for persons who had received the 2008–09 seasonal influenza vaccine, although it did not increase the odds for ARI.

Our study has some limitations, however. The uncertainty regarding the associated illness of pandemic (H1N1) 2009 at the time the study was initiated made the selection of a random sample not feasible. A pragmatic approach was therefore chosen to offer the entire school population the opportunity of being included in the study, resulting in 17% of the registered school population providing a serum sample. Because the study was conducted during an examination period, and some students and staff were absent, the size of the population from which the study sample was drawn is not known. However, because this was definitely less than the registered population, our response rate estimation was conservative. The distribution of our study population was not significantly different from the school’s population in age, school year, and sex (among teachers). However, the subsample of persons who provided a serum sample likely were not representative of persons who answered the questionnaire. For example, persons who provided a serum sample were more likely to have reported an ARI than persons who responded to the questionnaire only. This resulted in the overestimation of attack rates. Selection bias in the serology study subsample is also evident in the ORs for the 2008–09 seasonal influenza vaccine, for which the ORs were different, according to whether a serologic or clinical outcome was used, because the effect would be expected to be in the same direction. In addition, vaccination status was self-reported and could not be validated against official records, and we did not collect dates that antiviral drugs were used from each person.

We used a cutoff value of an HI titer >32 to indicate recent seroconversion. A previous study (*18*) has indicated that cross-reactive antibody to pandemic (H1N1) 2009 virus was prevalent in England’s population before the pandemic and that seroprevalence was strongly associated with age. In addition, a high proportion of children at the school had been vaccinated and were therefore unlikely to be representative of children in England. Conflicting evidence exists regarding the effect of prior trivalent influenza vaccination on cross-reactive titers for pandemic (H1N1) 2009 virus in persons <55 years of age (*21*,*24*). Therefore, some misclassification of cases (persons who seroconverted as a result of exposure to pandemic [H1N1] 2009 virus) likely occurred, leading to possible overestimation of the proportion of asymptomatic patients. However, the proportion of misclassified seropositive persons is likely small, particularly among children. Ideally, paired samples (collected before and after the outbreak) would have been able to measure seroconversion; however, this opportunity was not available.

These results highlight the fact that, depending on the virulence and transmissibility of an emerging influenza pandemic virus, extensive transmission may occur in a closed setting and thus by implication in the community over and above the observed clinical disease. This finding has notable implications for predicting the future course of a pandemic because the subsequent pool of those susceptible after initial transmission will diminish (*18*). Current policy in closed settings in the United Kingdom is to isolate or place symptomatic persons in cohorts after diagnosis to minimize the risk for onward transmission. If a substantial proportion of mildly symptomatic or even possibly asymptomatic persons were able to transmit infection, current policy would be of limited value. First, infection may be widespread within an institution long before it becomes apparent to public health authorities; second, a large number of persons may be infected but asymptomatic or mildly symptomatic when the first case is diagnosed. Although conclusions can be drawn from this study that rapid transmission of influenza occurred in this environment and that infection may not always produce symptoms, the evidence of transmission of the influenza virus by asymptomatic persons remains scant (*25*).

Our findings indicate that the use of prophylactic antiviral agents lowers the odds of an ARI by ≈50% but has no effect on reducing the odds of serologic infection. Several interpretations of these findings are possible; for example, while prophylactic antiviral agents might not reduce the risk for infection, they could protect from clinical disease. Published evidence from the occurrence of seasonal influenza has indicated that timely administration of prophylactic antiviral agents to close contacts of infected persons reduces the risk for disease (*26*). However, our results should be viewed with caution. First, the serology sample was a much smaller subset of the questionnaire survey respondents, and the results for a serologic outcome indicate a lack of power. Thus, the effectiveness of antiviral prophylaxis would be underestimated. Also, the low specificity of the case definition for ARIs could lead to an overestimation of the effect on clinical disease.

The association between self-reported illness and severity of illness with an increasing likelihood of seropositivity suggests that even in facilities with a limited diagnostic capacity, simple definitions of ILI may be a more specific indicator of the true presence of infection in communities in which a proven outbreak is under way. Fever was an essential part of the clinical case criteria for testing in the United Kingdom, in contrast to the United States, where the clinical criteria were either a “respiratory illness” (recent onset of >2 of the following: rhinorrhea/nasal congestion, sore throat, cough, fever or feverishness) or an ILI (fever >37.8°C [100°F], plus cough or sore throat).

This study has demonstrated evidence of widespread infection with pandemic (H1N1) 2009 virus in a closed setting, with a substantial proportion of asymptomatic persons. Although the study highlights the difficulties of obtaining a large representative sample from a boarding school population during a pandemic influenza outbreak, it also illustrates the value of such rapid field epidemiologic investigations in understanding an emergent threat. This was particularly relevant during the emergence of pandemic (H1N1) 2009 virus at a time when its pathogenicity was uncertain and the benefit of using antiviral agents for postexposure prophylaxis was unclear. The results of this seroepidemiologic study in an outbreak setting during the pandemic of novel pandemic (H1N1) 2009 highlight the need for health authorities to agree on protocols for similar investigations during future pandemics.

## Appendix

**Table A1:** Association of demographic characteristics, self-reported illness variables, and interventions with symptoms of ARI or ILI in boarding school population during outbreak of pandemic (H1N1) 2009, England*

Demographic characteristics and interventions	No. (%)† with completed questionnaire, n = 695	No. (%)† with completed questionnaire and matched sample, n = 353
Category		
Students	469 (67.5)	216 (61.2)
Staff	226 (32.5)	137 (38.8)
Age group, y		
13–15	219 (32.8)	90 (25.5)
16–18	250 (37.5)	126 (35.7)
20–49	111 (16.6)	71 (20.1)
>50	87 (13.0)	66 (18.7)
Unknown	28	
Sex, staff only†		
Female	109 (48.2)	71 (58.8)
Male	117 (51.8)	66 (48.1)
Role, staff only‡		
Nonteaching	135 (61.4)	93 (68.4)
Teaching	85 (38.6)	43 (31.6)
Unknown	6	1
Self-reported illness		
ARI		
No	424 (64.1)	199 (58.5)
Yes	237 (35.9)	141 (41.5)
Unknown	34	13
ILI, ARI plus fever		
No	551 (83.4)	277 (81.5)
Yes	110 (16.6)	63 (18.5)
Unknown	34	13
Severity§		
Mild	145 (62.8)	81 (57.9)
Moderate and severe	86 (37.2)	59 (42.1)
Unknown	6	1
Duration, d§		
1–2	26 (14.2)	14 (13.1)
3–7	88 (48.1)	52 (48.6)
7–10	33 (18.0)	18 (16.8)
>10	36 (19.7)	23 (21.5)
Unknown	54	34
Interventions		
Took antiviral drugs		
No	198 (33.5)	96 (31.7)
Yes	393 (66.5)	207 (68.3)
Unknown	104	50
Use of antiviral drugs: PEP vs. treatment doses		
PEP dose only	352 (59.6)	187 (61.7)
Treatment dose	41 (6.9)	20 (6.6)
No information on dose recorded	104	50
Completion of PEP course of antiviral drugs¶		
No antiviral drugs	198 (36.5)	96 (34.3)
Course not completed	51 (9.4)	25 (8.9)
Course completed	294 (54.1)	159 (56.8)
No information on completion of course provided	111	53
Seasonal influenza vaccine		
No	216 (33.7)	105 (31.3)
Yes	425 (66.3)	230 (68.7)
No information recorded	54	18

*ARI, acute respiratory infection; ILI, influenza-like illness; PEP, postexposure prophylaxis. †Percentage of total, excluding those categories in which the response was unknown or blank. ‡All students were male. §Of those who self-reported ARI: n = 237 with completed questionnaire; n = 141 with completed questionnaire and matched sample. ¶Excluding those who reported taking the treatment dose of antiviral drugs: n = 654 with completed questionnaire; n = 333 with completed questionnaire and matched sample.

## References

[1] Centers for Disease Control and Prevention. Swine influenza A (H1N1) infection in two children—southern California, March–April 2009. MMWR Morb Mortal Wkly Rep. 2009;58:1–4. 19390508

[2] Health Protection Agency and Health Protection Scotland New Influenza A(H1N1) Investigation Teams. Epidemiology of new influenza A (H1N1) in the United Kingdom, April–May 2009. Euro Surveill. 2009;14:pii:19213. 10.2807/ese.14.19.19213-en 19442403

[3] Kar-Purkayastha I, Ingram C, Maguire H, Roche A. The importance of school and social activities in the transmission of influenza A(H1N1)v: England, April–June 2009. Euro Surveill. 2009;14:pii:19311. 19712642 10.2807/ese.14.33.19311-en

[4] Health Protection Agency West Midlands H1N1v Investigation Team. Preliminary descriptive epidemiology of a large school outbreak of influenza A(H1N1)v in the West Midlands, United Kingdom, May 2009. Euro Surveill. 2009;14:pii:19264. 10.2807/ese.14.27.19264-en 19589329

[5] Sypsa V, Hatzakis A. School closure is currently the main strategy to mitigate influenza A(H1N1)v: a modeling study. Euro Surveill. 2009;14:pii: 19240. 19555599 10.2807/ese.14.24.19240-en

[6] Medical Officers of Schools Association. The MOSA handbook of school health, 18th ed. Stoke-on-Trent (UK): Trentham Books, Ltd; 1998.

[7] Smith A, Coles S, Johnson S, Saldana L, Ihekweazu C, O’Moore E. An outbreak of influenza A(H1N1)v in a boarding school in South East England, May–June 2009. Euro Surveill. 2009;14:pii:19263. 19589330 10.2807/ese.14.27.19263-en

[8] Brankston G, Gitterman L, Hirji Z, Lemieux C, Gardam M. Transmission of influenza A in human beings. Lancet Infect Dis. 2007;7:257–65. 10.1016/S1473-3099(07)70029-4 17376383

[9] Carrat F, Vergu E, Ferguson NM, Lemaitre M, Cauchemez S, Leach S, Time lines of infection and disease in human influenza: a review of volunteer challenge studies. Am J Epidemiol. 2008;167:775–85. 10.1093/aje/kwm375 18230677

[10] Monto AS, Koopman JS, Longini IM Jr. Tecumseh study of illness. XIII. Influenza infection and disease, 1976–1981. Am J Epidemiol. 1985;121:811–22. 4014174 10.1093/oxfordjournals.aje.a114052

[11] Skowronski DM, De Serres G, Crowcroft NS, Janjua NZ, Boulianne N, Hottes TS, Association between the 2008–09 seasonal influenza vaccine and pandemic H1N1 illness during Spring-Summer 2009: four observational studies from Canada. PLoS Med. 2010;7:e1000258. 10.1371/journal.pmed.1000258 20386731 PMC2850386

[12] Kelly H, Grant K. Interim analysis of pandemic influenza (H1N1) 2009 in Australia: surveillance trends, age of infection and effectiveness of seasonal vaccination. Euro Surveill. 2009;14:pii:19288. 19660248 10.2807/ese.14.31.19288-en

[13] Garcia-Garcia L, Valdespino-Gómez JL, Lazcano-Ponce E, Jimenez-Corona A, Higuera-Iglesias A, Cruz-Hervert P, Partial protection of seasonal trivalent inactivated vaccine against novel pandemic influenza A/H1N1 2009: case-control study in Mexico City. BMJ. 2009;339:b3928. 10.1136/bmj.b3928 19808768 PMC2758337

[14] Echevarria-Zuno S, Mejia-Aranguré JM, Mar-Obeso AJ, Grajales-Muñiz C, Robles-Pérez E, González-Leon M, Infection and death from influenza A H1N1 virus in Mexico: a retrospective analysis. Lancet. 2009;374:2072–9. 10.1016/S0140-6736(09)61638-X 19913290

[15] Effectiveness of 2008–09 trivalent influenza vaccine against 2009 pandemic influenza A (H1N1)—United States, May–June 2009. MMWR Morb Mortal Wkly Rep. 2009;58:1241–5. 19910912

[16] Rowe T, Abernathy RA, Hu-Primmer J, Thompson WW, Lu X, Lim W, Detection of antibody to avian influenza A (H5N1) virus in human serum by using a combination of serologic assays. J Clin Microbiol. 1999;37:937–43. 10074505 10.1128/jcm.37.4.937-943.1999 PMC88628

[17] Clark TW, Pareek M, Hoschler K, Dillon H, Nicholson KG, Groth N, Trial of 2009 influenza A (H1N1) monovalent MF59-adjuvanted vaccine. N Engl J Med. 2009;361:2424–35. 10.1056/NEJMoa0907650 19745215

[18] Miller E, Hoschler K, Hardelid P, Stanford E, Andrews N, Zambon M. Incidence of 2009 pandemic influenza A H1N1 infection in England: a cross-sectional serological study. Lancet. 2010;375:1100–8. 10.1016/S0140-6736(09)62126-7 20096450

[19] Wu JT, Ma ES, Lee CK, Chu DK, Ho PL, Shen AL, The infection attack rate and severity of 2009 pandemic H1N1 influenza in Hong Kong. Clin Infect Dis. 2010;51:1184–91. 10.1086/656740 20964521 PMC3034199

[20] Bandaranayake D, Huang QS, Bissielo A, Wood T, Mackereth G, Baker MG, Risk factors and immunity in a nationally representative population following the 2009 influenza A(H1N1) pandemic. PLoS ONE. 2010;5:e13211. 10.1371/journal.pone.0013211 20976224 PMC2954793

[21] Hardelid P, Andrews NJ, Hoschler K, Stanford E, Baguelin M, Waight PA. Assessment of baseline age-specific antibody prevalence and incidence of infection to novel influenza AH1N1 2009. Health Technol Assess. 2010;14:115–92. 21208549 10.3310/hta14550-03

[22] Zimmer SM, Crevar CJ, Carter DM, Stark JH, Giles BM, Zimmerman RK, Seroprevalence following the second wave of pandemic 2009 H1N1 influenza in Pittsburgh, PA, USA. PLoS ONE. 2010;5:e11601. 10.1371/journal.pone.0011601 20644650 PMC2904390

[23] Longini IM Jr, Koopman JS, Monto AS, Fox JP. Estimating household and community transmission parameters for influenza. Am J Epidemiol. 1982;115:736–51. 7081204 10.1093/oxfordjournals.aje.a113356

[24] Hancock K, Veguilla V, Lu X, Zhong W, Butler EN, Sun H, Cross-reactive antibody responses to the 2009 pandemic H1N1 influenza virus. N Engl J Med. 2009;361:1945–52. 10.1056/NEJMoa0906453 19745214

[25] Eccles R. Asymptomatic spread of flu is not proved. BMJ. 2005;331:1145. 10.1136/bmj.331.7525.1145 16282424 PMC1283287

[26] Welliver R, Monto AS, Carewicz O, Schatteman E, Hassman M, Hedrick J, Effectiveness of oseltamivir in preventing influenza in household contacts: a randomized controlled trial. JAMA. 2001;285:748–54. 10.1001/jama.285.6.748 11176912

